# Seroprevalence of the Hepatitis B, Hepatitis C, and Human Immunodeficiency Viruses and *Treponema pallidum* at the Beijing General Hospital from 2010 to 2014: A Cross-Sectional Study

**DOI:** 10.1371/journal.pone.0140854

**Published:** 2015-10-26

**Authors:** Shaoxia Xu, Qiaofeng Wang, Weihong Zhang, Zhifeng Qiu, Jingtao Cui, Wenjuan Yan, Anping Ni

**Affiliations:** 1 Department of Clinical Laboratories, Peking Union Medical College Hospital, Chinese Academy of Medical Sciences and Peking Union Medical College, Beijing, China; 2 Department of Infectious Diseases, Peking Union Medical College Hospital, Chinese Academy of Medical Sciences and Peking Union Medical College, Beijing, China; Alberta Provincial Laboratory for Public Health/ University of Alberta, CANADA

## Abstract

**Background:**

The hepatitis B, hepatitis C, human immunodeficiency viruses and *Treponema pallidum* are important causes of infectious diseases concern to public health.

**Methods:**

Between 2010 and 2014, we used an automated chemiluminescence microparticle immunoassay to detect the hepatitis B, hepatitis C, and human immunodeficiency viruses as well as *Treponema pallidum* (the rapid plasma regain test was used in 2010–2011). Positive human immunodeficiency virus tests were confirmed via western blotting.

**Results:**

Among 416,130 subjects, the seroprevalences for hepatitis B virus, hepatitis C virus, human immunodeficiency virus, and *Treponema pallidum* were 5.72%, 1.23%, 0.196%, and 0.76%, respectively. Among 671 patients with positive human immunodeficiency virus results, 392 cases were confirmed via western blotting. Hepatitis B and human immunodeficiency virus infections were more frequent in men (7.78% and 0.26%, respectively) than in women (4.45% and 0.021%, respectively). The hepatitis B and C virus seroprevalences decreased from 6.21% and 1.58%, respectively, in 2010, to 5.37% and 0.988%, respectively, in 2014. The human immunodeficiency virus seroprevalence increased from 0.04% in 2010 to 0.17% in 2014, and was elevated in the Infectious Disease (2.65%), Emergency (1.71%), and Dermatology and Sexually Transmitted Diseases (1.12%) departments. The specificity of the human immunodeficiency virus screening was 71.4%. The false positive rates for the *Treponema pallidum* screening tests increased in patients who were 60–70 years old. The co-infection rates for the hepatitis C and human immunodeficiency viruses were 0.47% in hepatitis C virus-positive patients and 7.33% in human immunodeficiency virus-positive patients.

**Conclusions:**

During 2010–2014, hepatitis B virus and human immunodeficiency virus infections were more frequent among men at our institution. Although the seroprevalences of hepatitis B and C viruses decreased, the seroprevalence of human immunodeficiency virus infection increased (with higher seroprevalences in high-risk departments). Older patients were more likely to exhibit false positive findings for syphilis.

## Introduction

The hepatitis B virus (HBV), hepatitis C virus (HCV), human immunodeficiency virus (HIV), and *Treponema pallidum* (TP) are blood-borne pathogens that are responsible for important infectious diseases. For example, it was reported that 240 million people were chronically infected with HBV and 150 million were chronically infected with HCV on World Hepatitis Day (July 28, 2014) [1]. Therefore, the World Health Organization (WHO) and its partners have urged policy-makers, healthcare workers, and the public to reconsider the importance of this silent killer [1]. Furthermore, during 2008, it was estimated that approximately 36.4 million adults worldwide were infected with syphilis [2]. Moreover, the HIV epidemic in China has gradually increased, and it was estimated that 780,000 people were infected with HIV during 2011 [3]. Unfortunately, the transmission of these infectious agents can involve various routes, including vertical transmission (from mother to infant), sexual contact, exposure to blood, and/or the transfusion of blood products. Therefore, hospitals are a common setting for transmission, both from patient to hospital staff (or vice versa) and from patient to patient.

The WHO also recommends comprehensive screening for all blood donations, using a quality control system to screen for HBV, HCV, HIV, and syphilis [4]. Thus, Chinese hospitals have implemented routine and mandatory screening tests for these infections, which take place before elective surgeries, invasive endoscopies, hospitalization, and blood transfusions and at pregnant women’s first antenatal visits. Therefore, we report our experience regarding the seroprevalences of HIV, HBV, HCV, and TP among Chinese individuals who were screened at the Peking Union Medical College Hospital (PUMCH) in Beijing, China. Using these findings, we aimed to evaluate the significance of this testing at one of China’s largest general hospitals.

## Subjects and Methods

### Ethics statement

The PUMCH Ethics Committee approved this retrospective study’s design (reference no.: SK-011) and waived the requirement for informed consent, as the 3-5mL of blood from each subject were collected for routine medical purposes and the subjects’ medical records were de-identified before the analysis.

### Study population

Between January 2010 and June 2014, we conducted a cross-sectional analysis of 416,130 Chinese inpatients, outpatients, and healthy individuals who were screened at PUMCH, in order to estimate the seroprevalences and risk factors that were associated with HBV, HCV, HIV, and TP infection. Our institution is a large general hospital (1,800 beds) that includes various departments and clinics, which provide medical care to people in Beijing and throughout China.

### Laboratory testing

A blood sample of 3–5 mL was drawn from each subject using a sterile Vacutainer (Becton-Dickinson Vacutainer System) for routine medical purposes, and the serum was separated via centrifugation. The serum specimens were then screened for the HBV surface antigen (HBsAg), anti-HCV antibodies, and HIV antigens/antibodies (Ag/Ab) using an automated chemiluminescence microparticle immunoassay (CMIA; Abbott i2000 SR). Based on the manufacturer’s instructions, positive results were recorded when the signal-to-cutoff ratio of the serum specimen was ≥1.0, and negative results were recorded for a signal-to-cutoff ratio of <1.0. For anti-HCV antibodies, all serum specimens with signal-to-cutoff ratio equal or more 1 but less than 5 in Abbott i2000 SR system were confirmed by Vitros 3600 system, Ortho Clinical Diagnostics, a Johnson & Johnson. Western blotting (MP Biomedicals Asia Pacific Pte Ltd.) was used to confirm positive screening results for HIV Ag/Ab. During 2010 and early 2011, the serum specimens were tested for TP using the rapid plasma regain test (RPR) (Shanghai Kehua Bio-engineering Co., Ltd.). However, from the latter part of 2011 to 2014, the CMIA was used to test the serum samples for TP (as well as HBV, HCV, and HIV).

### Statistical analysis

All data were coded, entered, sorted, and analyzed in Microsoft Excel 2007 (Microsoft Corp., New York, NY); p-values of <0.05 were considered statistically significant. For convenience, the age for pediatric patients (in days and months) was rounded off to the nearest year. The chi-square test was used to compare seroprevalences according to patient age, sex, hospital department, and year.

## Results

### Characteristics of the subjects

The subjects’ characteristics are shown in Table 1. A total of 416,130 Chinese individuals were screened between January 2010 and June 2014; 39.3% (n = 163,548) were men and 60.7% (n = 252,582) were women. The subjects’ ages ranged from one day after birth to 108 years (mean age, 45.6 ± 16.9 years; median age, 45 years).

**Table 1:** Characteristics of Chinese Subjects who were Screened at Peking Union Medical College Hospital (2010–2014, n = 416,130).

Variable		Mean age	No. of individuals	%
Sex	Male	47.5	163,548	39.3
	Female	44.2	252,582	60.7
Status	Outpatient	44.3	291,233	70.0
	Inpatient	48.1	124,897	30.0
Age (years)	0–10	N/A	5,975	1.4
	11–20	N/A	14,485	3.5
	21–30	N/A	66,719	16.0
	31–40	N/A	86,453	20.8
	41–50	N/A	83,456	20.1
	51–60	N/A	74,797	18.0
	61–70	N/A	48,779	11.7
	71–80	N/A	29,055	7.0
	>81	N/A	6,411	1.5
Study year	2010	46.0	72,449	17.4
	2011	45.7	85,433	20.5
	2012	45.2	93,811	22.5
	2013	45.1	108,095	26.0
	2014 (Jan–June)	45.6	56,342	13.5
Department	Internal Medicine	48.2	126,949	30.5
	Surgery	48.6	100,435	24.1
	Obstetrics & Gynecology	40.2	59,263	14.2
	Pregnancy^a^	31.4	17,673	4.2
	Pediatrics	12.8	3,118	0.75
	Infectious Disease & Hepatitis Clinic	41.1	8,456	2.0
	Dermatology & Sexually Transmitted Diseases	39.4	5,737	1.4
	Neurology	44.5	4,597	1.1
	EOS^b^	47.6	35,371	8.5
	Emergency	45.4	6,979	1.7
	Health examination	41.9	21,881	5.3
	Others^c^	49.2	25,671	6.2

^a^Pregnancy included normal pregnancies, including multiple gestations. Women with an ectopic pregnancy, stillbirth, or miscarriage were classified under Obstetrics & Gynecology.

^b^EOS: Departments of Eye, Otolaryngology, and Stomatology.

^c^Others: This category included geriatric patients, very important people, cases involving traditional Chinese medicine, political or goverment officials, and hospital staff.

#### Seroprevalences of HBV, HCV, HIV, and TP

The average seroprevalences of HBV, HCV, HIV, and TP during the study period were 5.72% (22,453/392,211), 1.23% (3,698/301,584), 0.196% (671/343,073), and 0.76% (1,904/250,997), respectively. Among the patients who were tested for TP, 0.53% (225/42,721) was defined as seropositive via the RPR test and 0.81% (1,679/208,051) was defined as seropositive via the CMIA. Of the 671 patients (0.196%) who initially screened positive for HIV, 392 (0.114%) cases were confirmed positive via western blotting, 157 (0.046%) were confirmed negative, 112 (0.033%) were undetermined, and 10 (0.003%) were lost to follow-up. When we excluded undetermined cases and those lost to follow-up, the average specificity of the CMIA for HIV was 71.4%, although the specificity varied according to subject characteristics and the treatment department. Sex-specific differences in the seroprevalences of HBV, HCV, HIV, and TP were relatively large, especially regarding the seroprevalences of HBV and HIV (Table 2). The seroprevalences of HBV and HCV decreased from 6.21% and 1.58%, respectively, in 2010, to 5.37% and 0.988%, respectively, in 2014. However, the prevalence of confirmed HIV infection increased by nearly 4.5-fold over the study period, from 0.038% in 2010 to 0.17% in 2014.

**Table 2:** Seroprevalences of Hepatitis B Virus, Hepatitis C Virus, Human Immunodeficiency Virus, and *Treponema pallidum* at Peking Union Medical College Hospital (2010–2014).

	HBsAgpos no. (%)	Anti-HCVpos no. (%)	HIV screeningpos no. (%)	HIV confirmed pos no. (%)	HIV CMIA specificity^a^	TPpos no. (%)^b^
Male	11,695 (7.78)	1,625 (1.46)	482 (0.366)	347 (0.26)	85.5	789 (0.875)
Female	10,758 (4.45)	2,073 (1.09)	189 (0.0895)	45 (0.021)	31.5	1,115 (0.69)
*χ* ^*2*^-value	1,908.17	79.93	315.42	413.85		25.08
*P*-value	<0.001	<0.001	<0.001	<0.001		<0.001
Outpatient	16,478 (6.13)	2,396 (1.29)	548 (0.24)	354 (0.16)	78.1	978 (0.72)
Inpatient	5,975 (4.84)	1,302 (1.12)	123 (0.11)	38 (0.033)	39.6	926 (0.81)
2010	4,282 (6.21)	710 (1.58)	55 (0.095)	22 (0.038)	57.9	153 (0.47)
2011	4,571 (5.72)	857 (1.52)	137 (0.195)	75 (0.11)	70.8	324 (0.77)
2012	5,024 (5.77)	801 (1.19)	161 (0.21)	101 (0.13)	77.7	663 (1.25)
2013	5,666 (5.55)	887 (1.00)	193 (0.21)	116 (0.13)	71.6	505 (0.62)
2014	2,910 (5.37)	443 (0.988)	125 (0.27)	78 (0.17)	69.4	259 (0.61)
Internal Medicine	7,325 (6.2)	1,202 (1.69)	191 (0.20)	95 (0.10)	65.1	433 (0.84)
Surgery	5,257 (5.3)	963 (1.0)	80 (0.084)	27 (0.03)	44.3	575 (0.68)
Obstetrics & Gynecology	2,258 (3.86)	311 (0.61)	39 (0.077)	6 (0.012)	20.0	317 (0.70)
Pregnancy	86 (2.8)	22 (0.86)	9 (0.053)	0	0	16 (0.65)
Pediatrics	73 (2.4)	9 (0.33)	1 (0.038)	0	0	8 (0.35)
Infectious Disease & Hepatitis Clinic	3,407 (33.7)	305 (6.70)	110 (3.27)	89 (2.65)	92.7	35 (1.98)
Dermatology & Sexually Transmitted Diseases	108 (3.7)	29 (1.13)	72 (1.35)	62 (1.12)	92.5	41 (1.73)
Neurology	231 (5.3)	48 (1.2)	10 (0.24)	1 (0.024)	14.3	71 (2.1)
EOS	1,412 (4.1)	227 (0.88)	51 (0.15)	25 (0.073)	59.5	151 (0.78)
Emergency	526 (8.3)	177 (3.67)	88 (1.93)	78 (1.71)	97.5	37 (0.87)
Health examination	867 (5.9)	102 (1.75)	6 (0.058)	3 (0.029)	75.0	28 (0.43)
Others	903 (3.5)	303 (1.2)	14 (0.055)	6 (0.02)	54.5	192 (0.75)
Total	22,453 (5.72)	3,698 (1.23)	671 (0.196)	392 (0.114)	71.4	1,904 (0.76)

^a^HIV CMIA specificity (%): western blot-confirmed HIV-positive cases divided by CMIA HIV-positive cases (excluding undetermined cases and cases lost to follow-up).

^b^TP screening was performed using the rapid plasma regain test (2010–2011) and the CMIA (2011–2014).

Pos, positive; HBsAg, hepatitis B surface antigen; HCV, hepatitis C virus; HIV, human immunodeficiency virus; CMIA, chemiluminescence microparticle immunoassay; TP, *Treponema pallidum*; EOS, Departments of Eye, Otolaryngology, and Stomatology; Others, geriatric patients, very important people, cases involving traditional Chinese medicine, political or goverment officials, and hospital staff.

**Fig 1:**
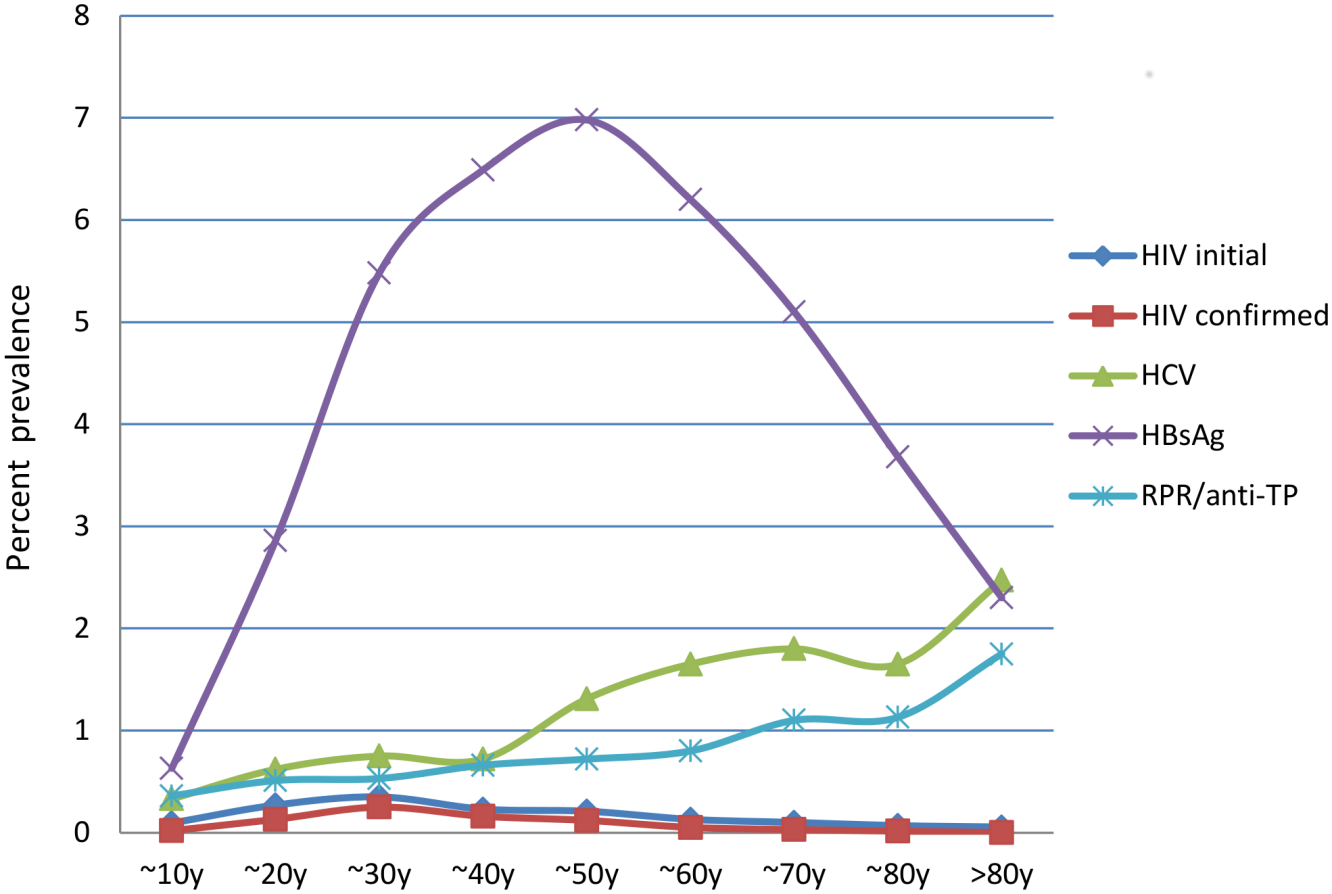
Seroprevalences of HBV, HCV, HIV, and TP According to Age. HBV, hepatitis B virus; HCV, hepatitis C virus; HIV, human immunodeficiency virus; TP, *Treponema pallidum*; RPR, rapid plasma regain test.

We observed a curved seroprevalence distribution for HBV according to age, with the highest prevalence (6.98%) observed in patients who were 41–50 years old (Fig 1). The 6.98% of HBsAg was significantly higher than the average prevalence (5.72%) of the studied population (χ^2^ = 185.4, p<0.001). In contrast, the seroprevalences of HCV and TP increased with age. The seroprevalence of HIV also increased with age, from 0.094% (positive screening) and 0.019% (confirmed cases) in subjects who were 0–10 years old, to 0.35% (positive screening) and 0.25% (confirmed cases) in subjects who were 21–30 years old. However, the seroprevalence of HIV subsequently decreased to 0.055% (positive screening) and 0% (confirmed cases) in subjects who were >80 years old.

**Fig 2:**
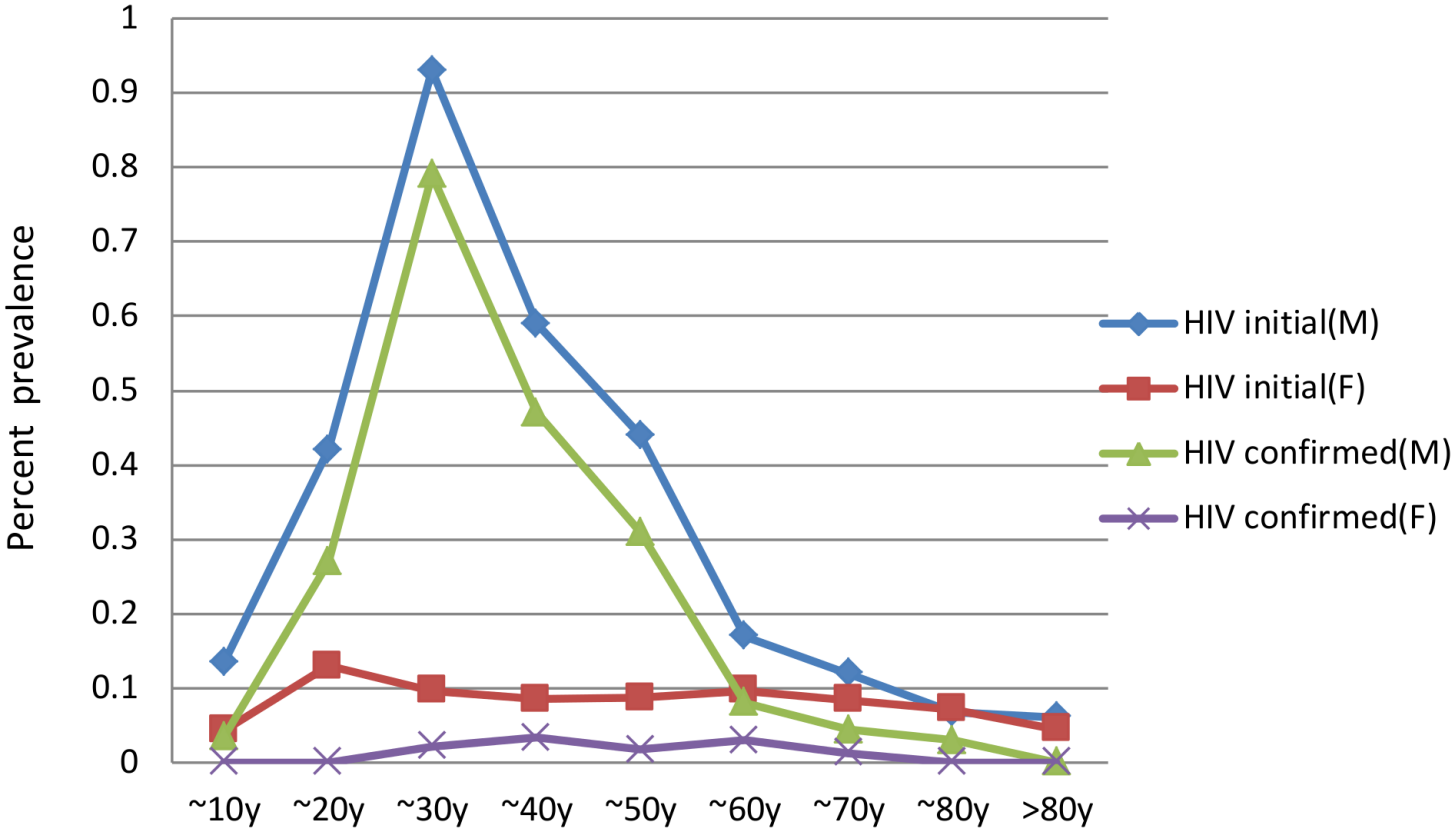
Sex-related Differences in the Seroprevalence of HIV According to Age. HIV, human immunodeficiency virus; M, male; F, female.

The sex-related differences in the seroprevalence of HIV according to age are shown in Fig 2. The seroprevalence of HIV infection in men (both positive screening and confirmed cases) increased during the teenage years, and peaked at the ages of 21–30 years, in which the prevalence was statistically higher than the average of total subjects (0.929% vs.0.196% for screening, χ^2^ = 358.8, p<0.001; 0.786% vs.0.114% for confirmed, χ^2^ = 475.0, p<0.001). However, after the age of 60 years, the seroprevalences of HIV in men were similar to the seroprevalences among women.

**Fig 3:**
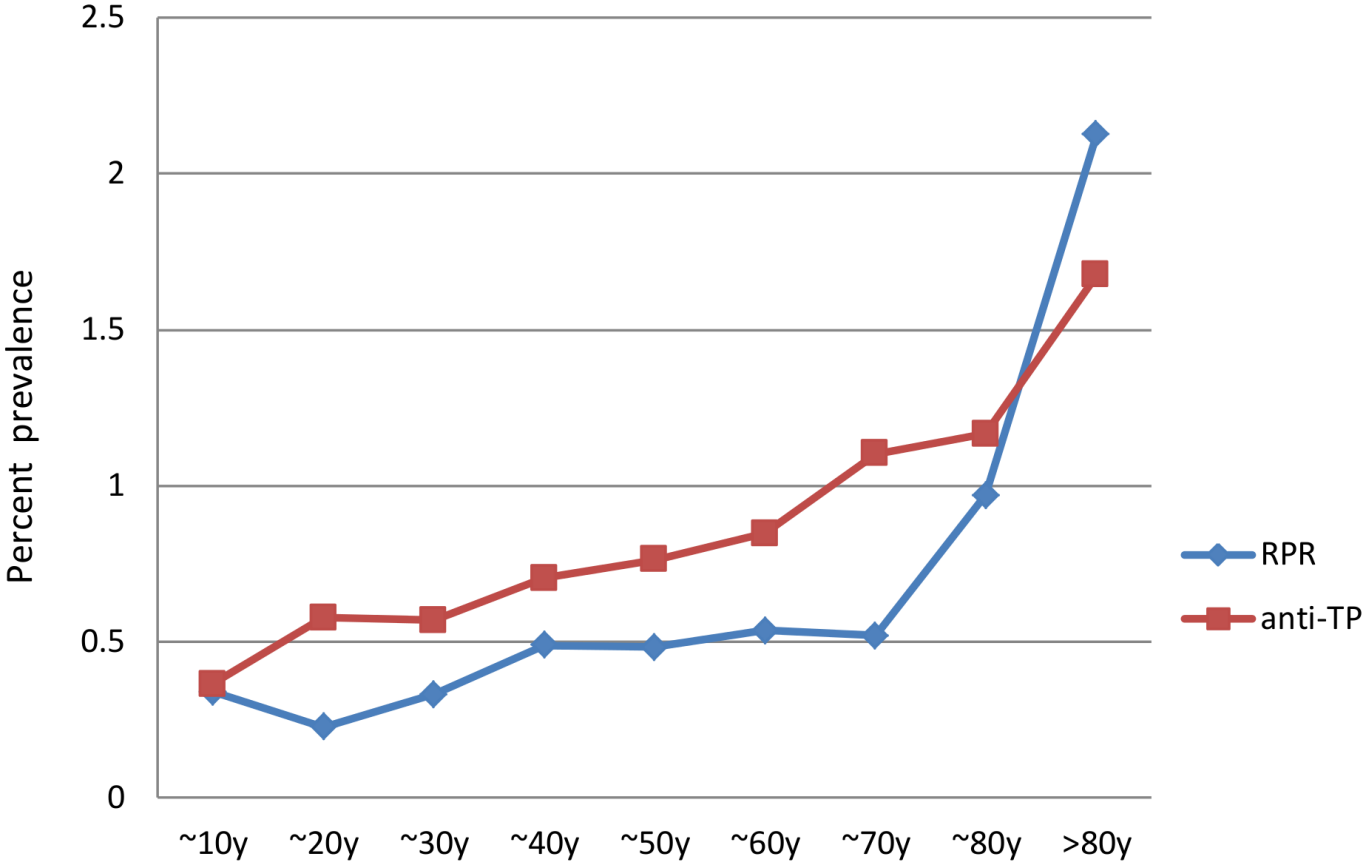
Seroprevalence of TP via the RPR Test and anti-TP According to Age. TP, *Treponema pallidum*; RPR, rapid plasma regain test.

The seroprevalences of TP, based on the RPR test (n = 42,721) and anti-TP (n = 208,051), according to age are shown in Fig 3. The anti-TP recorded a slightly higher seroprevalence at most ages (compared to the RPR test), although the RPR test recorded a slightly higher seroprevalence at ages >80 years old. The seroprevalence of RPR in subjects who were ≤ or >60 years old were 0.448% and 0.822%, respectively, the difference was statistically significant (χ^2^ = 18.1, p<0.001). Also, the seroprevalence of anti-TP in subjects who were ≤ or >60 years old were 0.71% and 1.17%, respectively, the difference was also statistically significant (χ^2^ = 91.6, p<0.001).

### Co-infection rates for HBV, HCV, and HIV

The co-infection rates for HBV, HCV, and HIV are presented in Table 3, although the TP seroprevalence data were excluded, because of the presumably high false positive rates in the subjects who were >60 years old. Among subjects who were HBV positive, 0.93% exhibited co-infection with HCV. Among subjects who were HCV-positive, 4.20% exhibited co-infection with HBV. These co-infection rates were lower than the total average prevalences of HCV (1.23%) and HBV (5.72%) infection. In contrast, among subjects who were HCV positive, 0.47% exhibited co-infection with HIV. Among confirmed HIV-positive subjects, 7.33% exhibited co-infection with HCV. These co-infection rates were higher than the average seroprevalences of confirmed HIV (0.114%) and HCV (1.23%) infection.

**Table 3:** Co-infection Rates for HBV, HCV, and HIV According to Sex.

	Men positive no. (%)	Women positive no. (%)	Ratio^a^ M/F	Total positive no. (%)
Co-infection of HBV with HCV	68 (1.18)	45 (0.70)	1.7:1	113 (0.93)
Co-infection of HCV with HBV	68 (5.65)	45 (3.02)	1.9:1	113 (4.20)
Co-infection of HBV with HIV(C)^b^	10 (0.16)	1 (0.015)	10.7:1	11 (0.085)
Co-infection of HIV(C) with HBV	10 (7.46)	1 (3.85)	1.9:1	11 (6.88)
Co-infection of HCV with HIV(C)	9 (0.88)	2 (0.15)	5.9:1	11 (0. 47)
Co-infection of HIV(C) with HCV	9 (7.09)	2 (8.70)	0.81:1	11 (7.33)
Co-infection of HBV with HCV and HIV(C)	2(1.75)	0	N/A	2 (1.48)

^a^Ratio (M/F): the ratio of co-infection in men and women.

^b^HIV(C): HIV-positive cases that were confirmed via western blotting.

M, male; F, female; HBV, hepatitis B virus; HCV, hepatitis C virus; HIV, human immunodeficiency virus.

## Discussion

Previous studies have reported that the prevalence of HBV was 9.8% in the general Chinese population and that 9–12% of children who were <5 years old had an HBV infection before the introduction of a nationwide HBV vaccination program, which was implemented in 1992 [5]. However, the prevalence of HBV has subsequently decreased to 7.2% among the general population in 2006, to 2.1% among all children who were born between 1992 and 2005, and to 1.0% among children who were born after 1999 [6,7]. Interestingly, we also observed a decrease in the seroprevalence of HBV, from 6.2% in 2010 to 5.4% in 2014. Furthermore, we found an average seroprevalence of 0.63% for HBV in children who were <10 years old, which is similar to the reported prevalence of 0.71% for HBV among hospitalized patients who were <16 years old (in Zhejiang Province, southeast China) [8]. Moreover, the curved distribution of HBV seroprevalences according to age was similar to the findings of previous reports [9,10]. This curved distribution is likely due to the fact that middle-aged subjects are least likely to have been immunized for HBV (a peak prevalence of 7% was observed among subjects who were 41–50 years old), and that older patients are increasingly likely to die due to HBV-related cirrhosis and hepatocellular carcinoma [11].

The rapid global spread of HCV infection is primarily due to efficient transmission via blood transfusion and exposure to contaminated medical equipment [12]. Based on the national epidemiological survey of viral hepatitis (1992–1995), the average prevalence of HCV was 3.2% in the general Chinese population [13]. Furthermore, the pooled prevalence of HCV infection before 1998 was 12.87% among blood donors, although it subsequently decreased to 1.71% after 1998 [14]. In our study, the average seroprevalence of HCV was 1.23% among all subjects, although the seroprevalence of HCV was 1.75% among subjects who underwent an annual health examination, and these subjects are likely more representative of the general adult population in Beijing. Interestingly, the seroprevalence of HCV decreased from 1.58% in 2010 to 0.988% in 2014, which may be due to the Blood Donation Law, which was enacted in 1998 and shifted blood donation from paid to voluntary donations. In addition, China introduced national regulation to prevent nosocomial infections during 2006, which might also take effort to reduce the prevalence of HCV. Nevertheless, as we did not follow-up the subjects who were HCV seropositive, it would be difficult to determine if the elevated HCV seroprevalence according to age was due to greater exposure to HCV or to false reactions that were caused by aging.

The previously reported prevalence of HIV infection at general hospitals in eight Chinese cities was 0.17% [15]. This prevalence was similar to our average seroprevalence (0.196%), which was determined via the CMIA screening test. In the present study, 21–30-year-old men had the highest seroprevalence of HIV (0.8%), and the prevalence of confirmed HIV infection increased steadily from 0.038% in 2010 to 0.17% in 2014. Epidemics of HIV infection in China focus on three populations, injecting drug users, female sex workers and men who have sex with men (MSM). HIV prevalence was stable in the first two populations, but it increased from 1.77% in 2000 to 5.98% in 2010 in MSM [16], and even higher prevalence was found in MSM in Eastern China (11.3%) [17] and in Tianjin city (12.4%) which was only 137 km from Beijing [18]. MSM sought free HIV testing through blood donation screening [19], or they also might seek HIV testing in our hospital. So, we presumed that the high HIV prevalence in 21–30-year-old men was highly associated with MSM. The reported prevalence of HIV infection among pregnant women was 0.66% in Brazil during 2014 [20], 0.38% in India during 2013 [21], 0.1% in Spain during 2006 [22], and 0.01–0.02% in Zhejiang Province (eastern China was low) during 2007–2013 [23]. In contrast, we found that only 0.053% of pregnant women were HIV seropositive at the initial screening test, and none of these cases were confirmed to be HIV positive via western blotting. This finding appears to indicate that the vertical transmission of HIV is limited at our institution. However, the reported prevalence of HIV infection among 4,366,283 Chinese blood donations was 0.088% (2000–2010) [5], and we also observed that the seroprevalence of HIV in individuals for health examinations was 0.0558% (6/10,286) during our initial screening, although the prevalence of HIV decreased to 0.029% when we only considered confirmed cases (via western blotting).

The Abbott Architect i1000SR HIV Ag/Ab Combo assay has a reported specificity of 81.25% in a low incidence (0.64%) population [24]. However, we found that the specificity of this assay was low among low-risk subjects, who included all female subjects, pregnant women, and patients who sought care in the Obstetrics and Gynecology and Pediatrics departments. Nevertheless, the assay’s specificity was >90% in high-risk subjects, especially among patients who visited the Infectious Disease and Hepatitis Clinic (92.7%), Dermatology and Sexually Transmitted Diseases (92.5%), and Emergency (97.5%) departments. This finding is likely because there is a relatively high incidence of HIV infection in these departments. In addition, the overall specificity of the HIV CMIA increased from 57.9% in 2010 to 70.8% in 2011 (Table 2), which may be related to the assay’s updated HIV-Ab reagent, which was introduced during 2011.

Chen et al. [25] have reported a resurgence of syphilis in China during the past 20 years, and another Chinese study [26] has found that there was a significant growth in the serological markers of syphilis among first-time Chinese blood donors (2008–2010). In contrast, we did not observe a serious syphilis epidemic, as the overall seroprevalence of TP was only 0.76%, and the seroprevalence was only 1.73% among patients who visited the Dermatology and Sexually Transmitted Diseases clinic. However, as syphilis is a sexually transmitted disease, it is difficult to explain the increased seroprevalence of TP that we observed among subjects who were >60 years old. One possible explanation for this observation is that false positive reactions increase with age (i.e., interference). This potential interference would be relevant in clinical practice, as physicians and surgeons at our institution (and possibly other institutions) prefer to avoid elective operations, endoscopies, or pacemaker installations if the patient is seropositive for TP.

The association between HBV and HCV infections may be related to their shared transmission routes, especially blood transfusion. In addition, the estimated prevalences of HBV and HCV co-infection are approximately 5–20% among HBV-positive patients and 2–10% among HCV-positive patients, although these prevalences vary widely according to geographical location [27]. For example, Liaw et al. have reported that the HBV and HCV co-infection rate was 12% among HBV-positive patients in Taiwan [28], although Tyson et al. [29] have reported that the HBV and HCV co-infection rate was only 1.3% among a large sample of American HCV-positive patients. In contrast, we found that the HBV and HCV co-infection rates among HBV- or HCV-positive subjects were similar to the average seroprevalences in the general study population. However, HIV co-infection influences the clinical outcome of patients with an HCV infection by accelerating progression to advanced liver disease [30]. Similarly, we also found that HIV and HCV co-infection was more common among patients who were HCV-positive or who had a confirmed HIV infection.
